# The UCSC Ebola Genome Portal

**DOI:** 10.1371/currents.outbreaks.386ab0964ab4d6c8cb550bfb6071d822

**Published:** 2014-11-07

**Authors:** Maximilian Haeussler, Donna Karolchik, Hiram Clawson, Brian J Raney, Kate R. Rosenbloom, Pauline A. Fujita, Angie S. Hinrichs, Matthew L Speir, Chris Eisenhart, Ann S. Zweig, David Haussler, W. James Kent

**Affiliations:** CBSE, University of California Santa Cruz, Santa Cruz, California, USA; CBSE, University of California Santa Cruz, Santa Cruz, California, USA; CBSE, University of California Santa Cruz, Santa Cruz, California, USA; CBSE, University of California Santa Cruz, Santa Cruz, California, USA; CBSE, University of California Santa Cruz, Santa Cruz, California, USA; CBSE, University of California Santa Cruz, Santa Cruz, California, USA; Genomics Institute, University of California Santa Cruz, Santa Cruz, California, USA; CBSE, University of California Santa Cruz, Santa Cruz, California, USA; CBSE, University of California Santa Cruz, Santa Cruz, California, USA; CBSE, University of California Santa Cruz, Santa Cruz, California, USA; CBSE, University of California Santa Cruz, Santa Cruz, California, USA

**Keywords:** ebola, ebolavirus, EBOV, genome analysis, genomics

## Abstract

Background: With the Ebola epidemic raging out of control in West Africa, there has been a flurry of research into the Ebola virus, resulting in the generation of much genomic data. Methods: In response to the clear need for tools that integrate multiple strands of research around molecular sequences, we have created the University of California Santa Cruz (UCSC) Ebola Genome Browser, an adaptation of our popular UCSC Genome Browser web tool, which can be used to view the Ebola virus genome sequence from GenBank and nearly 30 annotation tracks generated by mapping external data to the reference sequence. Significant annotations include a multiple alignment comprising 102 Ebola genomes from the current outbreak, 56 from previous outbreaks, and 2 Marburg genomes as an outgroup; a gene track curated by NCBI; protein annotations curated by UniProt and antibody-binding epitopes curated by IEDB. We have extended the Genome Browser’s multiple alignment color-coding scheme to distinguish mutations resulting from non-synonymous coding changes, synonymous changes, or changes in untranslated regions. Discussion: Our Ebola Genome portal at http://genome.ucsc.edu/ebolaPortal/ links to the Ebola virus Genome Browser and an aggregate of useful information, including a collection of Ebola antibodies we are curating.

## Introduction

The Ebola epidemic continues to grow in West Africa. The U.S. Centers for Disease Control (CDC) estimated the occurrence of 21,000 cases in Sierra Leone and Liberia alone by Sept. 30, 2014, surging to 1,400,000 cases by Jan. 20, 2014, if the epidemic continues to grow at the current pace^1^. Against such a backdrop, research on Ebola antibodies and vaccines is a high priority. Much of the research on the current epidemic involves genomic sequencing of the virus, including three genomes from Guinea^2^ and 99 genomes from Sierra Leone^3^. Sequence annotations are available from established database curation teams: UniProt^4^ has manually annotated the protein sequences and the Immune Epitope and Analysis Resource^5^ has collected epitope sequences from previously published studies. These diverse datasets can all be mapped to the genome sequence. However, existing tools such as the NCBI Virus Genome Browser^6^ and the Viral Genome Organizer^7^ show only gene models. VIPR^22^ is a toolset to annotate sequences but the results are not available instantly and does not merge them into an integrated zoomable view. Reasoning that the University of California Santa Cruz (UCSC) Genome Browser^8^^,^ 9 could be adapted quickly to help with analysis of the current outbreak, we built an Ebola Genome Browser that aggregates a wide range of data from sources worldwide.

The UCSC Genome Browser is a mature web tool for rapid and reliable display of any requested portion of a genome at any scale. The genome itself forms the horizontal axis that can be zoomed and scrolled. The vertical axis is a stack of annotation tracks, each containing a particular type of data. Examples of common annotation track types for a typical vertebrate genome include genes, comparative multiple alignments of many genomes, and SNPs. The tracks can be displayed at various levels of detail, and clicking on an item in a track displays a page of information about that item.

We have adapted the Genome Browser to support the display of the Ebola virus genome and a diverse set of annotations. In addition to the Ebola Genome Browser, we constructed an Ebola Portal page that wraps around the browser and other collected resources. These include a set of sequences of antibodies that bind Ebola, for use in research into vaccines and antiserum type therapies and links to many other Ebola resources.

## Materials and Methods

We started with the UCSC Genome Browser code base, primarily written in C, which includes utilities for transforming data from one format to another, tools for loading the MySQL database, and CGI programs that create web pages based on the contents of the database. The source code, available at https://genome-store.ucsc.edu/, is free for academic and non-profit use, but requires licensing for commercial use.

The UCSC Genome Browser displays centers around a reference genome assembly to which all annotations are aligned. After conversations on the compatibility of annotations with Dr. Pardis Sabeti from the Broad Institute, we decided to use the sequence from GenBank accession KM034562.1 as our reference sequence. This allowed us to quickly import the extensive set of 99 Ebola genomes from Gire et al. (2014)^3^ without reformatting. We next ran our multiz pipeline^10^ on the viral genomes to align them to the reference sequence, and used UCSC tools to add information from the GenBank gene annotation. We wrote various text-processing utilities to import data from UniProt^4^, the Immune Epitope Database (IEDB)^5^ and the Protein Data Bank (PDB)^11^, and used HMMER3^12^ to align protein domain models from Pfam^13^ against the NCBI Ebola gene set. All tracks were described using the Genome Browser trackDb system^14^.

## Results

**An Ebola reference genome browser**

We have successfully produced a UCSC Genome Browser on the Ebola virus genome (Figure 1). The multiple-alignment display was extended specifically for Ebola to show the effects of nucleotide mutations on viral proteins (non-synonymous, synonymous, stop, etc.). The close evolutionary distance among different Ebola sequences is readily observable in this new display, where only one or two coding SNPs across the entire genome are seen between two isolates from different patients in the same outbreak.

**UCSC Ebola Genome Browser:**
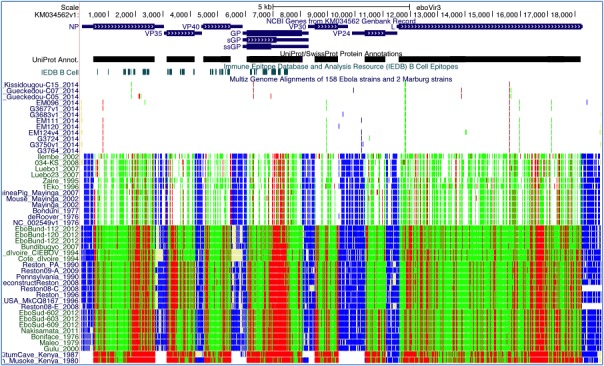
Four annotation tracks are displayed. The NCBI Genes track follows a display convention in which coding regions are shown at full height and UTRs at half height. Clicking on an item in this track takes the user to a page of information on the gene. The UniProt/SwissProt Protein Annotations track is shown in dense display mode. Clicking on this track expands it such that individual items, including protein motifs, domains, cleavage sites and other features, are labeled and staggered vertically if they overlap, as is done in the gene track. The B-Cell Epitopes track from IEDB, displayed in dense mode, shows protein regions where antibodies are known to bind. The Multiz Genome Alignments track is configured to show just a printable subset of the available genomes. When zoomed out to view the full genome (as here), changes between the reference genome are visible as colored lines: green for synonymous coding changes, red for non-synonymous, blue for UTR changes, and light yellow for missing data. When zoomed in sufficiently, individual amino acid and base differences are shown instead.

**Checking the impact of the Sierra Leone 2014 mutations on an epitope**

Across various strains of Ebola and various outbreaks dating back to 1976, the crucial GP gene, which is the only one exposed on the surface of the virus, has large regions that are conserved. When combined with the positive results in non-human primate trials, it seems likely that the vaccines now in Phase I trials^15^^,^ 16 and previously developed antibodies will be effective across a large range of Ebola viruses.

Figure 2 illustrates how one can use the Genome Browser to inspect the degree of variation of a particular antibody epitope sequence in the current outbreak. At the top of the display, the browser shows the region of the Ebola genomic nucleotide reference sequence in view, here zoomed to a 77 bp region. The UniProt track indicates that one is looking at a part of the GP1 protein, in the extracellular, mucin-like region, located next to a CHZ (histone chaperone) domain (shown in the Pfam track).

**UCSC Ebola Genome Browser zoomed in to show the epitope of the 4G7 antibody, part of ZMapp, a triple antibody experimental Ebola treatment:**
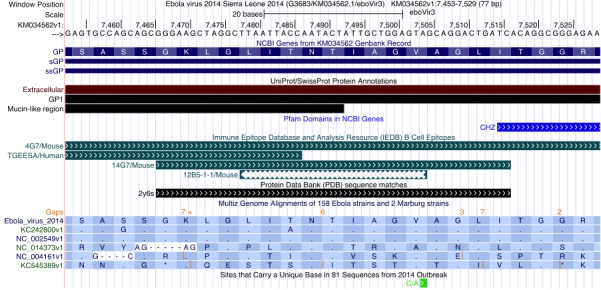
In this view the NCBI Genes, SwissProt, Pfam, IEDB and PDB annotation tracks have been set to “pack” display mode, which shows descriptive labels of the individual track features. The Multiz Genome Alignments annotation showing the alignment of other Ebola strain sequences to the reference sequence has been configured by setting the 160 Accessions track to “full” display mode, which (at this zoom level) shows amino acid sequences for each of the strains listed. In this example, the number of displayed strains has been reduced to only one sequence per outbreak by adjusting additional track settings, accessed by clicking on the 160 Accessions track label. The annotation “Sites that Carry a Unique Base…” is displayed by setting the 2014 Specific variation track to “pack” mode.

The IEDB track in Figure 2 shows that the protein encoded by the nucleotide sequence in this view is partially targeted by four previously published antibodies: an unnamed one from a screen (TGEESA), 12B5-1-1, 14G7 and 4G7. Detailed information about each of these antibodies can be viewed by clicking on the antibody name in the display. In this instance, the details reveal that 14G7 and 4G7 were first described by Olal et al. (2012)^17^ and Qiu et al. (2011)^18^, respectively. Comparing the IEDB and PDB tracks, note that the 14G7 IEDB feature maps to the same region as the 2y6s feature in the PDB track: a crystal structure of the antibody 14G7 is available in the Protein Data Bank under accession 2y6s. A click on the 2y6s feature in the display will show this structure and a link to the primary citation. Olal et al. note that the most significant residues for 14G7 antibody binding are TIAG. Note that there is no matching PDB track feature for the 4G7 antibody: a crystal structure for that antibody, part of the experimental ZMapp drug^19^, is not available but one can observe that it is binding the same sequence.

The Multiz Genome Alignments annotation in Figure 2 has been configured to show amino acid sequences, with only one strain sequence from each outbreak displayed: Sierra Leone 2014 (translation of the reference sequence), Zaire 07 (KC242800), Zaire 76 (NC_002549), Bundibugyo 07 (NC_014373), Reston 89 (NC_004161) and Sudan 76 (KC545389). In this annotation, a dot indicates that the amino acid is identical to that of the reference sequence at the same position. As previously shown in this journal^20^^,^ 21, the Sierra Leone sequence most resembles the two Zaire sequences. This is supported by rows 2 and 3 in the Multiz Genome Alignment display, which show mostly dots relative to the reference sequence in row 1.

As shown in the bottom annotation in Figure 2, the 2014 outbreak sequence does have a mutation in this region (C/A), but the green color of the feature indicates that it is synonymous. Therefore, both the 14G7 and 4G7 (ZMapp) antibodies most likely will be effective against the current 2014 strain if they were effective against the original Zaire strain.

This example, which can be repeated with any of the other curated 77 IEDB epitopes in other regions of the virus, shows how one can use the Genome Browser to get a quick overview of available information across various databases and determine whether the data support a given hypothesis. In addition to the data natively displayed in the UCSC Genome Browser, users can import their own annotation data for display on the reference sequence using the browser’s custom track feature (http://genome.ucsc.edu/goldenPath/help/customTrack.html).

**The UCSC Ebola Genome Portal**

We link to the UCSC Ebola Genome Browser from a portal page (http://genome.ucsc.edu/ebolaPortal/) that contains references to numerous other resources and a collection of sequences for antibodies against Ebola that we are curating in support of vaccine and antiserum developers.

## Discussion

Given the exponential growth rate of the virus and the mobility of the human population, the current Ebola virus outbreak may not be not contained until late 2015. Today’s research efforts may provide some help in managing this outbreak; therefore, anything that can be done to encourage Ebola research and data-sharing seems prudent. To this end we hope the UCSC Ebola Portal and Genome Browser will be a useful tool for researchers. Suggestions for additional tracks and general feedback may be sent to the UCSC Genome Browser public mailing list at genome@soe.ucsc.edu.
